# An acute vesiculobullous eruption in newly diagnosed systemic lupus erythematosus

**DOI:** 10.1016/j.jdcr.2026.09.045

**Published:** 2026-09-30

**Authors:** Leena Wardeh, Kayla Brockmeyer, Angela Zaladonis, Shayan Waseh, Karan Pandher

**Affiliations:** Department of Dermatology and Cutaneous Biology, Thomas Jefferson University, Philadelphia, Pennsylvania

**Keywords:** autoimmune blistering disease, bullous lupus erythematosus, direct immunofluorescence (DIF), subepidermal blister, systemic lupus erythematosus (SLE), vesiculobullous eruption

## Case description

A 56-year-old woman with a newly diagnosed systemic lupus erythematosus (SLE) presented with the abrupt onset of painful blistering lesions. She had been diagnosed with SLE approximately 3 months earlier and was started on hydroxychloroquine 400 mg daily and a prednisone taper beginning at 40 mg daily. Within days of initiating therapy, she developed a new, painful, widespread blistering eruption.

Physical examination demonstrated widespread tense vesicles and bullae involving the face, oral mucosa, neck, trunk, and upper extremities, with scattered erosions of the hard palate (Fig 1, Fig 2, Fig 3).

**Fig 1:**
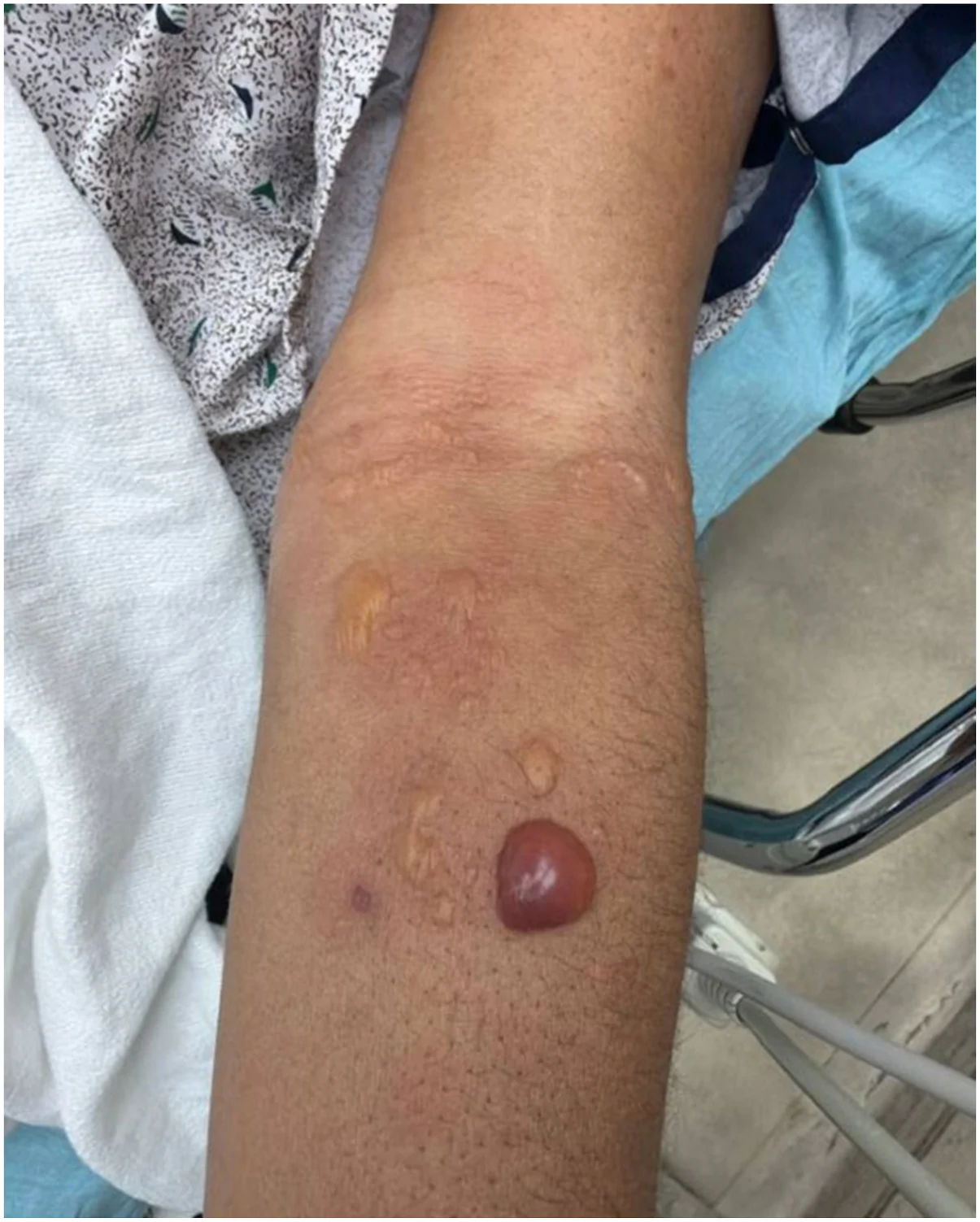
Tense vesicles and bullae with background erythema of the upper extremity.

**Fig 2:**
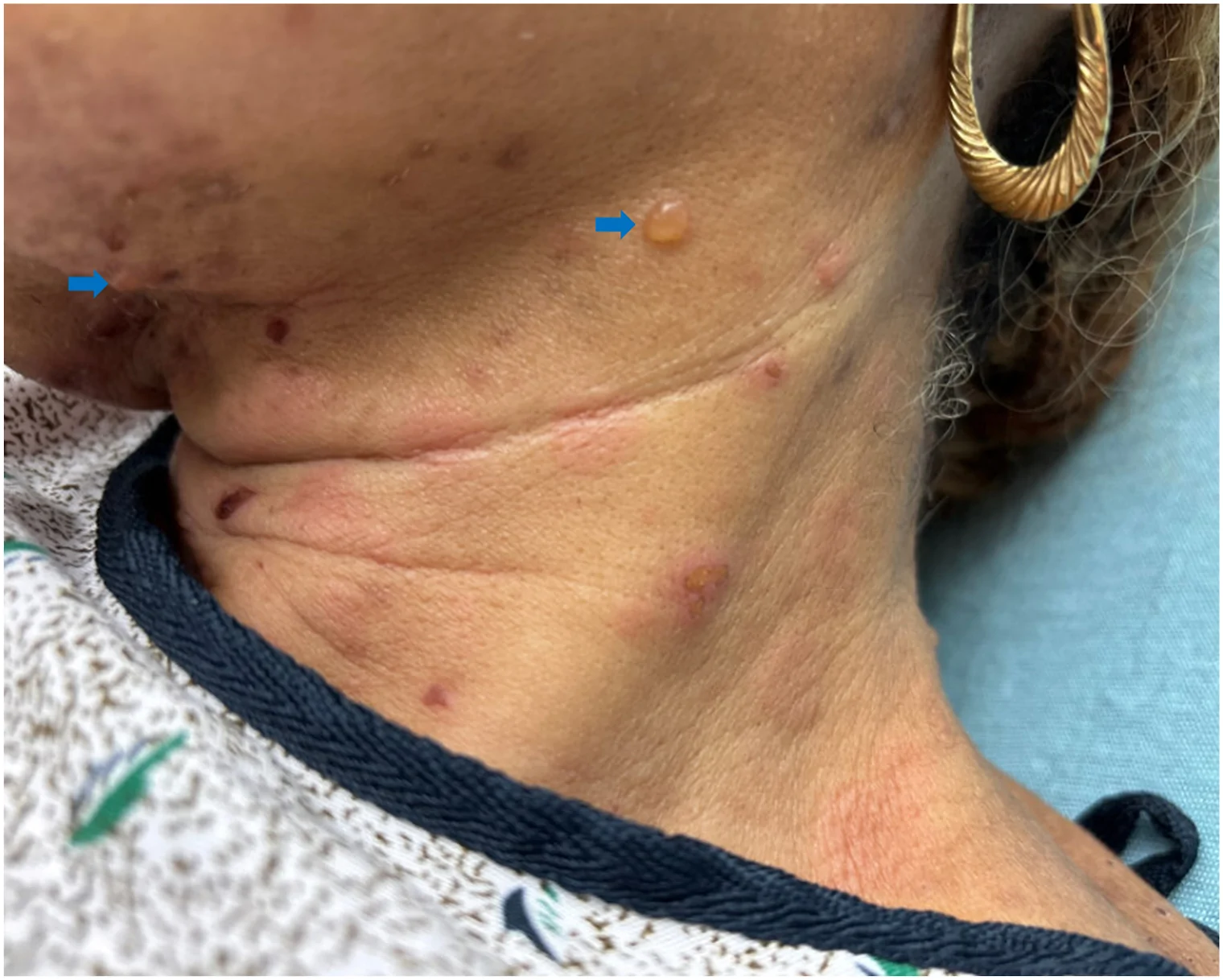
Vesicles (*blue arrows*), and erosions of the chin and neck.

**Fig 3:**
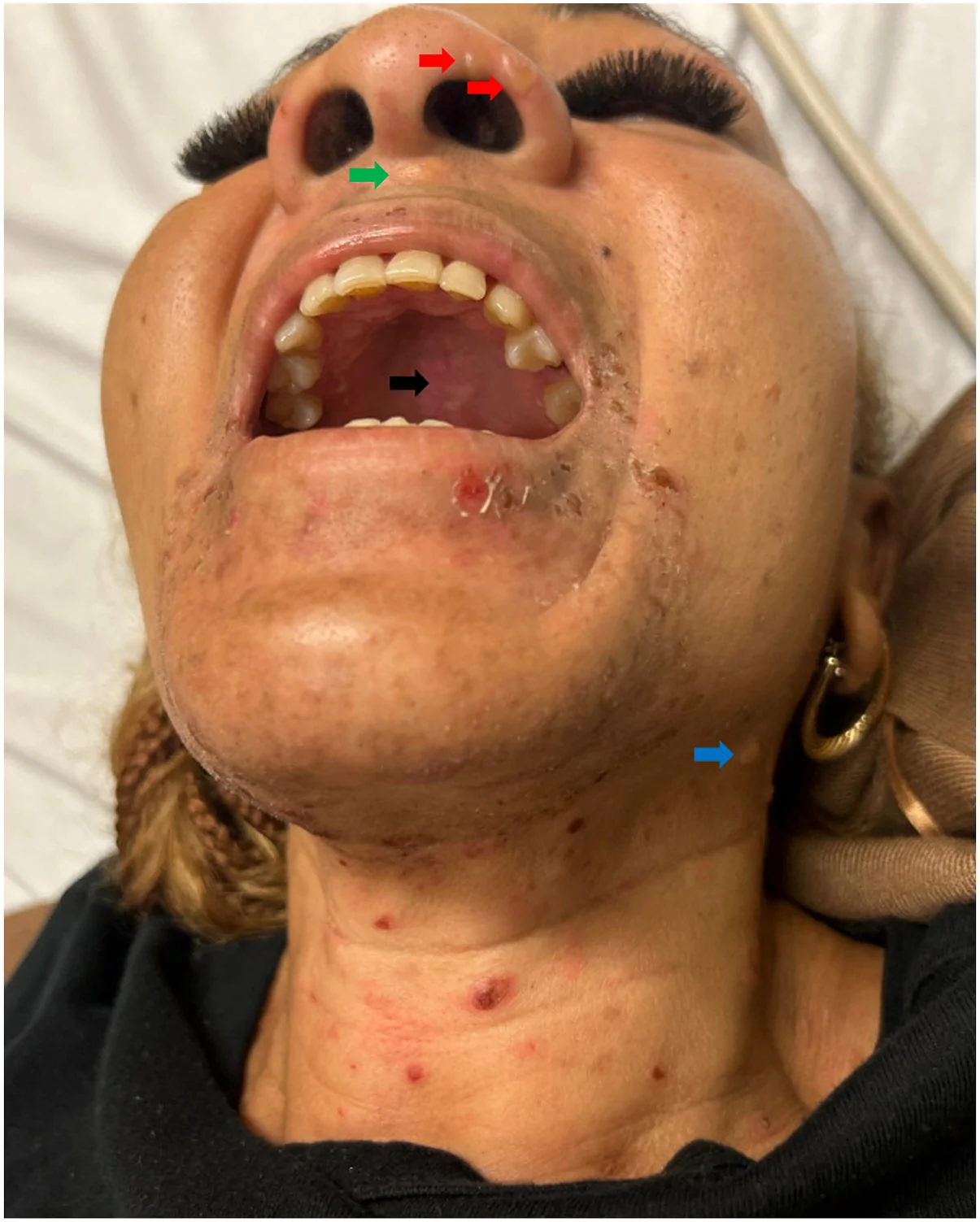
Tense bullae of left nasal ala (*red arrows*), cutaneous upper lip (*green arrow*), and neck (*blue arrow*). Erosions of the hard palate (*black arrow*). Erosions of perioral region, chin, and neck.

In a patient with SLE, blistering may arise from multiple mechanisms beyond primary autoimmune blistering disease, including bullous drug eruptions, vasculitis, and bullous change within acute cutaneous lupus lesions. Given the recent diagnosis of SLE, however, bullous SLE was favored over other subepidermal blistering dermatoses, including linear IgA bullous dermatosis, epidermolysis bullosa acquisita, and bullous pemphigoid. Punch biopsies of the left forearm were obtained for hematoxylin and eosin staining and direct immunofluorescence (DIF). Hematoxylin and eosin demonstrated a subepidermal blister with numerous neutrophils outlining the dermal-epidermal junction (Fig 4), and DIF revealed linear deposition of IgG, IgA, IgM, and C3 along the basement membrane zone (Fig 5). This clinicopathologic constellation confirmed the diagnosis of bullous SLE.

**Fig 4:**
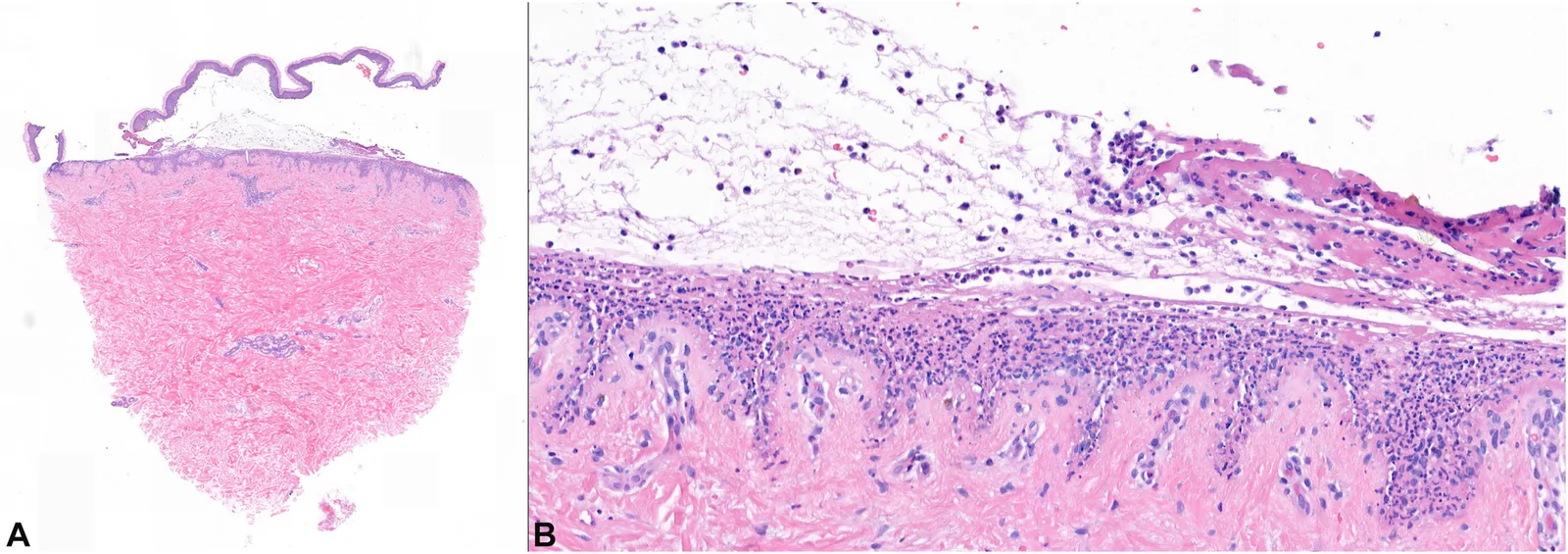
H&E sections of left forearm lesion. **A,** Low power (2×) view demonstrating subepidermal blister. **B,** Higher power (20×) view of subepidermal blister with numerous neutrophils which appear to be outlining the dermal-epidermal junction. *H&E*, Hematoxylin and eosin.

**Fig 5:**
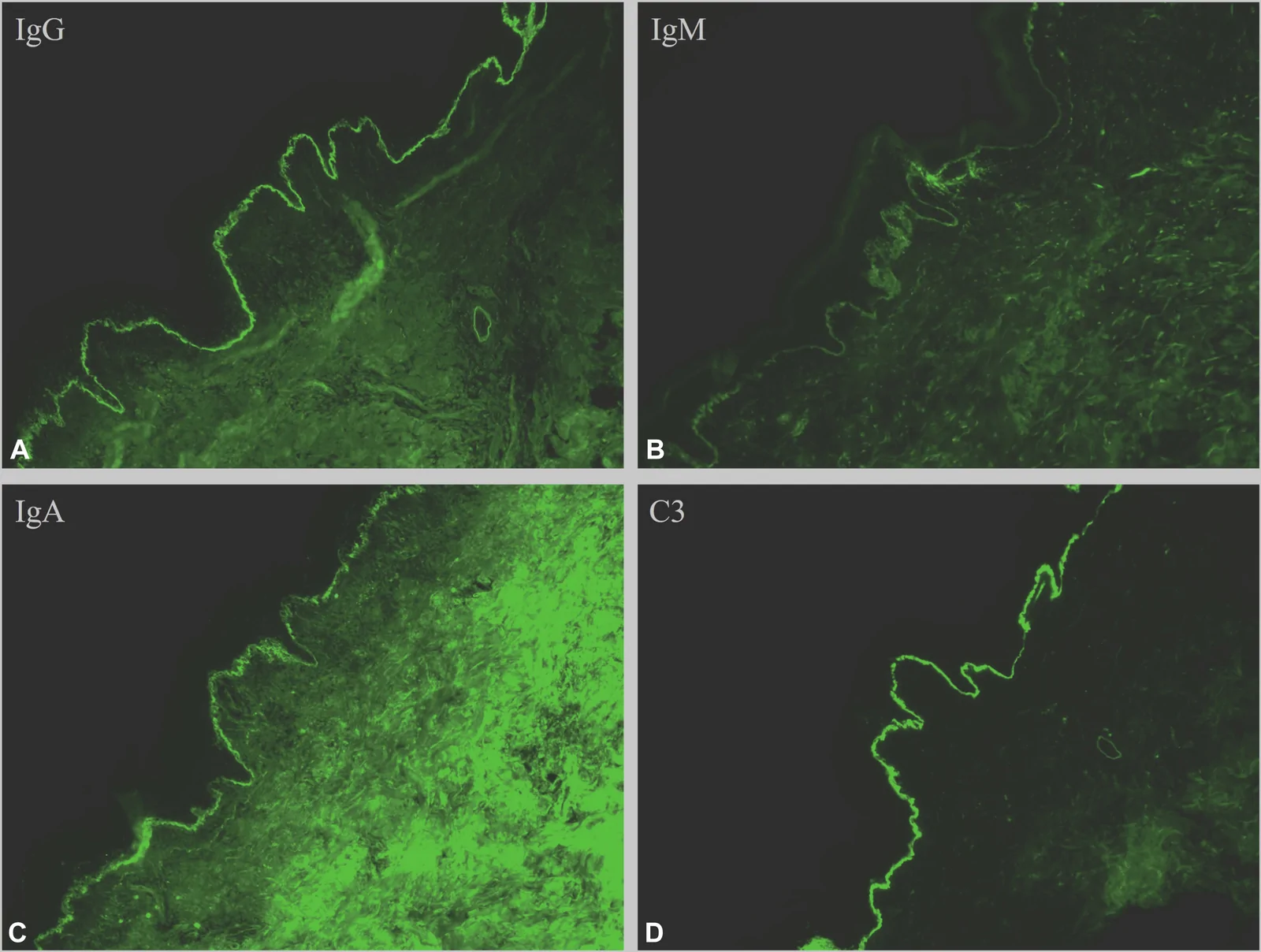
DIF demonstrating linear deposition along the basement membrane zone, including IgG **(A)**, IgA **(B)**, IgM **(C)**, and C3 **(D)**. *DIF*, Direct immunofluorescence.

The patient was started on dapsone 50 mg daily with significant improvement; at 10-day follow-up the dose was uptitrated to 100 mg daily for a few persistent vesicles, with subsequent resolution of all cutaneous symptoms.

**Question: Which of the following blistering conditions shares a common target antigen with bullous lupus erythematosus?**

**A.** Dermatitis herpetiformis
**B.** Epidermolysis bullosa acquisita
**C.** Bullous pemphigoid
**D.** Porphyria cutanea tarda
**E.** Pemphigus vulgaris

**Correct Answer**: **B.**

## Discussion

Bullous SLE is an autoantibody-mediated subepidermal blistering disease driven by autoantibodies against type VII collagen, the principal component of the anchoring fibrils that secure the dermal-epidermal junction. This is the same target antigen implicated in epidermolysis bullosa acquisita.^1^, ^2^ The two share an antigen but are distinguished by clinical context and serology rather than by their target: bullous SLE arises only in patients with established SLE, whereas epidermolysis bullosa acquisita occurs independently of lupus, is often cell-poor histologically, and is typically refractory to therapies effective in bullous SLE.^2^

Bullous SLE is rare, affecting fewer than 5% of patients with SLE, and predominates in women and patients of African descent.^2^, ^3^ Its onset often reflects active systemic disease; lupus nephritis, frequently class III or IV, has been reported in roughly half of cases, so a new bullous eruption should prompt assessment of disease activity and renal involvement.^3^ Mucosal lesions occur in up to half of patients and must be distinguished from the oral ulcers of SLE itself.^3^

Diagnosis rests on clinicopathologic correlation. Hematoxylin and eosin should be obtained from the edge of a new bulla, and DIF from erythematous, lesional skin. Because immunoglobulin deposition may be present in both lesional and clinically uninvolved skin, reflecting the lupus band phenomenon, DIF is a key diagnostic tool even when routine histology is nonspecific.^4^, ^5^ Indirect immunofluorescense on salt-split skin, demonstrating dermal-side binding, and enzyme-linked immunosorbent assay for anti-type VII collagen antibodies, are additional acceptable confirmatory methods.^2^ Dapsone is first-line therapy, and brisk clinical response supports the diagnosis. Screening for glucose-6-phosphate dehydrogenase deficiency is required before initiating dapsone to avoid hemolysis.^1^

## Conflicts of interest

None disclosed.
